# Whole exome sequencing revealed 14 variants in *NDP, FZD4*, *LRP5,* and *TSPAN12* genes for 20 families with familial exudative vitreoretinopathy

**DOI:** 10.1186/s12920-022-01204-0

**Published:** 2022-03-11

**Authors:** Handong Dan, Dongdong Wang, Zixu Huang, Qianqian Shi, Miao Zheng, Yuanyuan Xiao, Zongming Song

**Affiliations:** Henan Provincial People’s Hospital, Henan Eye Hospital, Henan Eye Institute, Henan Key Laboratory of Ophthalmology and Visual Science, People’s Hospital of Zhengzhou University, People’s Hospital of Henan University, No. 7 Weiwu Road, Zhengzhou, 450000 Henan China

**Keywords:** Familial exudative vitreoretinopathy, Whole exome sequencing, NDP, FZD4, LRP5, TSPAN12

## Abstract

**Background:**

Familial exudative vitreoretinopathy (FEVR) is a complex form of blindness-causing retinal degeneration. This study investigated the potential disease-causing variants in 20 Chinese families with FEVR.

**Methods:**

All available family members underwent detailed ophthalmological examinations, including best-corrected visual acuity and fundus examination. All probands and most family members underwent fluorescein fundus angiography. Twenty probands underwent whole exome sequencing; 16 of them also underwent copy number variant and mitochondrial genome analysis. Bioinformatics analysis and Sanger sequencing of available family members were used to confirm the disease-causing gene variant.

**Results:**

Twenty families were diagnosed with FEVR based on clinical symptoms, fundus manifestations, and fundus fluorescein angiography. Whole exome sequencing revealed 14 variants in *NDP, FZD4, LRP5,* and *TSPAN12* genes among the 13 families. These variants were predicted to be damaging or deleterious according to multiple lines of prediction algorithms; they were not frequently found in multiple population databases. Seven variants had not previously been reported to cause FEVR: c.1039T>G p.(Phe347Val) in the *FZD4* gene; c.1612C>T p.(Arg538Trp) and c.3237-2A>C in the *LRP5* gene; and c.77T>A p.(Ile26Asn), c.170dupT p.(Leu57Phe fsTer60), c.236T>G p.(Met79Arg) and c.550dupA p.(Arg184Lys fsTer16) in the *TSPAN12* gene. We did not detect any variants in the remaining seven families.

**Conclusions:**

These results expand the spectrum of variants in the *NDP, FZD4*, *LRP5,* and *TSPAN12* genes and provide insights regarding accurate diagnosis, family genetic counseling, and future gene therapy for FEVR.

**Supplementary Information:**

The online version contains supplementary material available at 10.1186/s12920-022-01204-0.

## Background

Familial exudative vitreoretinopathy (FEVR, OMIM: 133780) is a clinically and genetically heterogeneous inherited ophthalmic disorder [1, 2]. It is characterized by incomplete retinal vascular development and pathological neovascularization [3]. Patients usually complain of reduced visual acuity or blindness in early childhood. The fundus can exhibit peripheral retinal avascularization, falciform retinal folds, macular ectopia, retinal exudate, retinal neovascularization, and retinal detachment [4]. However, some patients may not complain of any visual impairment; they may only exhibit peripheral avascularization [5]. The reported prevalence is approximately 0.11% in newborns [6], but, the actual prevalence may be underestimated because some patients are asymptomatic and demonstrate peripheral retinal involvement only [7].

FEVR can be inherited in autosomal dominant, autosomal recessive, or X-linked manners; the most common mode of inheritance is autosomal dominant [8]. Thus far, the following eleven genes have been reported to cause FEVR: norrin (*NDP*, OMIM, 300658) [9], frizzled 4 (*FZD4*, OMIM, 604579) [10], low density lipoprotein receptor-related protein 5 (*LRP5*, OMIM, 603506) [11], tetraspanin 12 (*TSPAN12*, OMIM, 613310) [12], catenin beta 1 (*CTNNB1*, OMIM, 116806) [13], zinc finger protein 408 (*ZNF408*, OMIM, 616454) [14], atonal homolog 7 (*ATOH7*, OMIM, 609875) [15], kinesin family member 11 (*KIF11*, OMIM, 148760) [16], RCC1 and BTB domain containing protein 1 (*RCBTB1*, OMIM, 607867) [17], jagged 1 (*JAG1*, OMIM, 601920) [18], and α-catenin (*CTNNA1*, OMIM) [19]. Moreover, one locus, EVR3, which maps to 11p13-p12, can also lead to FEVR; its causative gene has not been fully identified [20]. Among these pathogenic genes, *FZD4*, *LRP5*, and *TSPAN12* are the most common disease-causing genes related to FEVR [21]. The first five genes are involved in the Norrin or Wnt/β-catenin signaling pathway and have functions in cell adhesion, migration, and signaling [22].

Although increasing numbers of gene variants have been identified using next generation sequencing technology, these reported gene variants are responsible for only 50–60% of FEVR cases. Moreover, some patients may exhibit rapid progression without correct diagnosis and intervention. Thus, it is imperative to ascertain genetic etiology and achieve accurate diagnosis for affected patients, especially patients who are asymptomatic and exhibit peripheral retinal involvement alone. In this study, 20 families were diagnosed with FEVR based on clinical manifestations. We performed whole exome sequencing of probands and Sanger sequencing of available family members to elaborate the underling disease-causing gene variant.

## Methods

### Clinical examinations

Detailed premature delivery history, oxygen uptake history, family history, and birth weight information were acquired for the probands. Exhaustive ophthalmological examinations were completed, including best-corrected visual acuity, intraocular pressure, slit-lamp microscopy, ophthalmoscopy, fundus photography, and fundus fluorescein angiography (FFA). All participants underwent pupillary dilation with a mixture of 0.5% phenylephrine hydrochloride and 0.5% tropicamide eye drops (Santen Pharmaceutical, Osaka, Japan). Fundus photography was performed with a VISUCAM 200 digital fundus camera (Carl Zeiss Meditec AG, Jena, Thuringia, Germany) or Optos Daytona ultrawide field system (Optos PLC, Dunfermline, United Kingdom). FFA was acquired using SPECTRALIS Engineering systems (Heidelberg Engineering Ltd, Hertfordshire, United Kingdom) or Optos Daytona ultrawide field system (Optos PLC) with 20% fluorescein solution (Guangzhou Pharmaceutical Holdings Limited, Guangzhou, China). FEVR was diagnosed based on previously reported criteria [23].

### Whole exome sequencing

Genomic DNA samples were prepared from peripheral blood of all participants using a TIANamp Genomic DNA Kit (TIANGEN Biotech, Beijing, China). Library construction was performed from proband samples using xGen Exome Research Panel (Integrated DNA Technologies, Coralville, Iowa, United States). Samples were sequenced on a HiSeq platform (Illumina, San Diego, California, United States) or MGISEQ-T7 platform (BGI Genomics, Shenzhen, Guangdong, China) using a whole exome sequencing protocol, in accordance with the manufacturer’s instructions. Sequence data were analyzed for corresponding ophthalmologic inherited genes, especially inherited retinal disease genes; sequences were aligned using Burrows-Wheeler Aligner (http://bio-bwa.sourceforge.net/). Variant calling and nomenclature complied with the recommendations of the Human Genome Variation Society (http://www.hgvs.org/). Variant annotation was performed in accordance with American College of Medical Genetics (ACMG, https://www.acmg.net/) guidelines.

### Raw reads filtering

First, quality control was carried out for the enriched genes to filter out reads that did not meet quality control criteria. After whole exome sequencing, raw reads were filtered to remove duplicates, then aligned to the hg19 (GRCh37) human genome reference sequence. Quality control was recalibrated by Picard Mark Duplicates (http://sourceforge.net/projects/picard/), Genome Analysis Toolkit (https://gatk.broadinstitute.org/hc/en-us), and SAM tools (http://samtools.sourceforge.net/). Variants were validated and analyzed preferentially if they met the following previously reported criteria [24–26]: (1) minor allele frequency of the variant < 0.01 in the 1000 Genomes Project database (http://www.internationalgenome.org/), Exome Aggregation Consortium database (http://exac.broadinstitute.org/), Genome Aggregation database (http://gnomad.broadinstitute.org/), and an in-house Chinese individuals database; (2) variant location in an exon region or canonical splicing intron region that affected transcription splicing; (3) damaging or deleterious variant prediction using Sorting Intolerant From Tolerant (http://sift.jcvi.org/), Protein Variation Effect Analyzer (http://provean.jcvi.org), Polymorphism Phenotyping (http://genetics.bwh.harvard.edu/pph2/), ClinPred (https://sites.google.com/site/clinpred/), Likelihood Ratio Test (http://www.genetics.wustl.edu/jflab/lrt), Mutation Taster (http://www.mutationtaster.org/), Mutation Assessor (http://mutationassessor.org/r3), and Functional Analysis Through Hidden Markov Models (http://fathmm.biocompute.org.uk/); (4) highly conserved variant prediction using Genomic Evolutionary Rate Profiling (http://mendel.stanford.edu/SidowLab/downloads/gerp); (5) other reported pathogenic variant that did not meet the above criteria (e.g., high minor allele frequency variant, deep-intronic variant, or synonymous single nucleotide variant).

### In silico analysis

Preferentially selected variants were validated and cosegregated by Sanger sequencing, performed using an 3500xL Dx Genetic Analyser (Applied Biosystems, Foster City, California, United States) with ABI BigDye Terminator v3.1 Cycle Sequencing kit (Applied Biosystems). Primers were designed with Primer3 (http://primer3.ut.ee/) to amplify *NDP, FZD4*, *LRP5,* and *TSPAN12* gene fragments. The primer information is provided in Additional file 1: Table S1. Consensus sequences corresponding to proband sequences were downloaded from national center for biotechnology information (https://www.ncbi.nlm.nih.gov/). All sequences were analyzed using SeqMan II software in the Lasergene software package (DNASTAR, Madison, Wisconsin, United States). Evolutionary conservation among different species for single nucleotide variants were analyzed using MegAlign software in Lasergene software package (DNASTAR). Genomic and protein structures were schematically represented using IBS 1.0 software (http://ibs.biocuckoo.org).

## Results

### Clinical manifestations

**Fig. 1:**
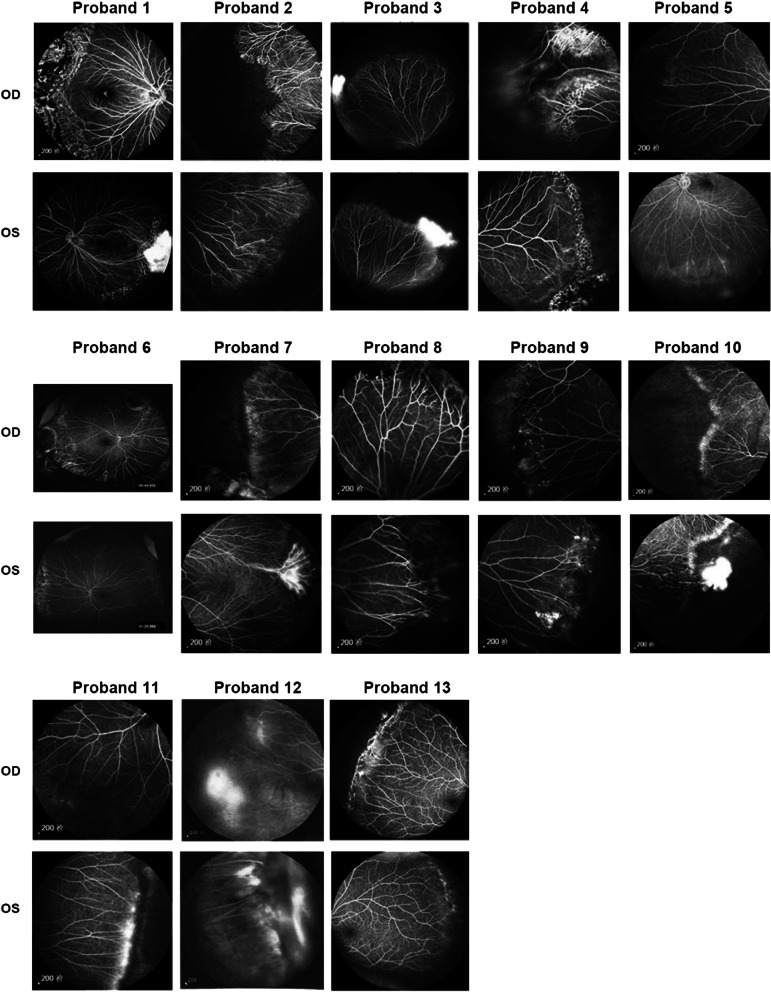
FFA manifestations of probands with potential disease-causing variants. FFA demonstrated non-perfusion areas, retinal leakage, retinal avascularization, straightened vessels, and increased numbers of vessels. Abbreviations: *OD* right eye, *OS* left eye

All participants had no history of premature delivery, problems with oxygen uptake, or low birth weight. They had no systematic complaints or extraophthalmic abnormalities that could be identified through conventional examinations (e.g., short stature or microcephaly). All probands complained of reduced visual acuity. Fundus examination of both eyes showed increased numbers of peripheral retinal vessels with willow-like retinal vessels. FFA demonstrated non-perfusion areas, retinal leakage, retinal avascularization, straightened vessels, and increased numbers of vessels. FFA manifestations of probands with potential disease-causing variants are presented in Fig. 1. The ophthalmic features of all probands with potential disease-causing variants are summarized in Table 1. Based on clinical manifestations, they were diagnosed with FEVR.

**Table 1:** Clinical features of all probands with potential disease-causing variants

ID	Gender	Symptom	Age at (year)		BCVA		Fundus examination		FFA	
			Onset	Exam	OD	OS	OD	OS	OD	OS
1	M	VD	13	15	0.2	0.8	IPRV, WLRS, LS	IPRV, WLRS, LS	IPRV, WLRS, LS, NPA	IPRV, WLRS, LS, NPA, PRA, PRL
2	M	VD, S	4	10	0.5	0.12	IPRV, WLRS	IPRV, WLRS	IPRV, WLRS, NPA	IPRV, WLRS, NPA, PRA, PRL
3	F	VD, S	4	5	0.4	0.1	IPRV, WLRS	IPRV, WLRS	IPRV, WLRS, NPA, PRA, PRL	IPRV, WLRS, NPA, PRA, PRL
4	M	VD, S	3	9	0.6	1.0	IPRV, WLRS, LS	IPRV, WLRS, LS, ME	IPRV, WLRS, LS, NPA, PRL	IPRV, WLRS, LS, ME, NPA
5	F	VD	35	50	1.0	0.6	IPRV, WLRS	IPRV, WLRS	IPRV, WLRS, NPA	IPRV, WLRS, NPA
6	F	VD	20	30	0.1	0.8	IPRV, WLRS, PoRD	IPRV, WLRS	IPRV, WLRS, PoRD, PRL	IPRV, WLRS, PRL
13	M	VD	21	21	0.01	0.3	IPRV, WLRS, PoRD, LS	IPRV, WLRS	IPRV, WLRS, NPA, PoRD, LS	IPRV, WLRS, NPA, PRA, PRL
8	M	VD, S	10	10	0.6	0.2	IPRV, WLRS, LS	IPRV, WLRS, LS	IPRV, WLRS, LS, NPA	IPRV, WLRS, LS, NPA, PRL
9	M	VD	24	33	0.6	0.6	IPRV, WLRS, LS	IPRV, WLRS, LS	IPRV, WLRS, LS, NPA, PRL	IPRV, WLRS, LS, NPA, PRL
10	M	VD, S	15	15	0.6	0.6	IPRV, WLRS	IPRV, WLRS	IPRV, WLRS, NPA	IPRV, WLRS, NPA, PRA, PRL
11	M	VD	30	34	FC	0.5	IPRV, WLRS	IPRV, WLRS	IPRV, WLRS, NPA, PRL	IPRV, WLRS, NPA, PRL
12	M	VD	18	24	1.0	0.8	IPRV, WLRS	IPRV, WLRS, ME, FRF, PRE, IPRV	IPRV, WLRS, NPA, PRA	IPRV, WLRS, ME, FRF, PRE, IPRV, NPA
13	F	VD, S	4	5	0.6	0.07	IPRV, WLRS	IPRV, WLRS	IPRV, WLRS, NPA	IPRV, WLRS, NPA

M, Male; F, Female; BCVA, best-corrected visual acuity; OD, right eye; OS, left eye; FFA, fluorescein fundus angiography; VD, vision decline; S, strabismus; IPRV, increased peripheral retinal vessels; WLRS, willow-like retinal vessels; FRF, falciform retinal folds; ME, macular ectopia; PRE, peripheral retinal exudates; PoRD, postoperative retinal detachment; LS, laser speckles; PRA, peripheral retinal avascularization; PRL, peripheral retinal leakage; NPA, No perfusion areas

### Sequencing and in silico analysis results

**Fig. 2:**
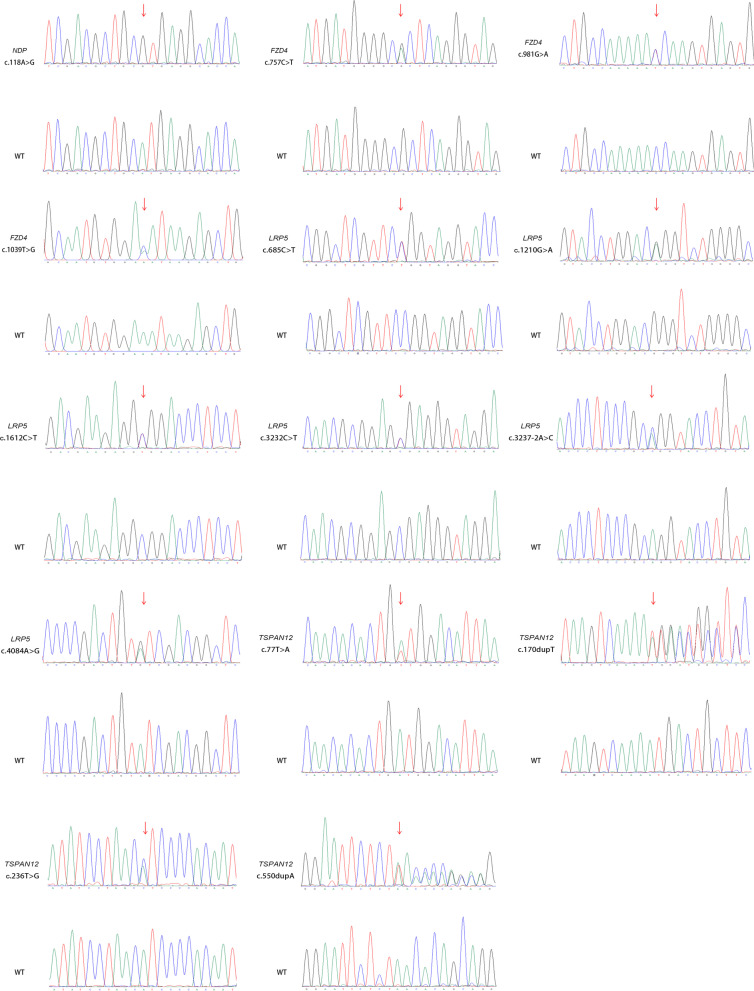
Sanger sequencing chromatographs of mutant and wild-type base for probands. Arrows denote mutant bases. Abbreviations: *WT* wild-type

After filtering, the cleaned data of all probands met the quality control criteria. The quality control data for the probands are summarized in Additional file 1: Table S2. In total, 20 probands underwent whole exome sequencing; 16 probands also underwent copy number variation and mitochondrial genome analysis. None of the 16 probands harbored any copy number variant or mitochondrial genome variant. Through bioinformatics analysis and Sanger sequencing, we detected 14 variants in the *NDP*, *FZD4*, *LRP5*, and *TSPAN12* genes among the 13 families; seven variants had not previously been reported to cause FEVR. We did not detect any variants in the remaining seven families. Variant c.981G>A in the *FZD4* gene did not cosegregate with the disease in family 3, possibly because it was a de novo variant or the proband’s biological parents exhibited chimerism. Because peripheral blood samples from family 5 were unavailable, we could not complete the cosegregation analysis. Sanger sequencing of the remaining 11 families revealed that the variants cosegregated with the FEVR phenotypes in these families. Sanger sequencing chromatographs of the variants are shown in Fig. 2. The pedigrees and cosegregation of families are presented in Additional file 1: Figure S1. Variant information for the *NDP, FZD4, LRP5,* and *TSPAN12* genes is summarized in Table 2. The variants were located in different exons or intron of the *NDP, FZD4, LRP5,* and *TSPAN12* genes; they were predicted to affect the various domains of the NDP, FZD4, LRP5, and TSPAN12 proteins. Schematic representations of the genomic and protein structures of all variants are shown in Additional file 1: Figure S2A and S2B.

**Table 2:** Information regarding variants in *NDP, FZD4, LRP5, and TSPAN12* genes of probands

ID	WES	CNV	MGS	Gene	Transcript	Nucleotide change	Amino acid change	Variant type	Exon	State	ACMG	Reference (PMID)
1	+	+	+	*NDP*	NM_000266	c.118A>G	p.(Met40Val)	missense	E2	Hem	UVS	30768221
2	+	+	+	*FZD4*	NM_012193	c.757C>T	p.(Arg253Cys)	missense	E2	Het	LP	30452590
3	+	+	+	*FZD4*	NM_012193	c.981G>A	p.(Trp327Ter)	nonsense	E2	Het	LP	30452590
4	+	−	−	*FZD4*	NM_012193	c.1039T>G	p.(Phe347Val)	missense	E2	Het	UVS	Novel
5	+	+	+	*LRP5*	NM_002335	c.685C>T	p.(Arg229Trp)	missense	E3	Het	LP	31589614
6	+	−	−	*LRP5*	NM_002335	c.1210G>A	p.(Gly404Arg)	missense	E6	Het	LP	16252235
6	+	−	−	*LRP5*	NM_002335	c.1612C>T	p.(Arg538Trp)	missense	E8	Het	LP	Novel
7	+	−	−	*LRP5*	NM_002335	c.3232C>T	p.(Arg1078Ter)	nonsense	E14	Het	LP	20340138
8	+	+	+	*LRP5*	NM_002335	c.3237-2A>C	−	splice	I14	Het	P	Novel
9	+	+	+	*LRP5*	NM_002335	c.4084A>G	p.(Ile1362Val)	missense	E19	Het	UVS	30097784
10	+	+	+	*TSPAN12*	NM_012338	c.77T>A	p.(Ile26Asn)	missense	E3	Het	UVS	Novel
11	+	−	−	*TSPAN12*	NM_012338	c.170dupT	p.(Leu57Phe fsTer60)	nonsense	E4	Het	LP	Novel
12	+	−	−	*TSPAN12*	NM_012338	c.236T>G	p.(Met79Arg)	missense	E4	Het	LP	Novel
13	+	+	+	*TSPAN12*	NM_012338	c.550dupA	p.(Arg184Lys fsTer16)	nonsense	E7	Het	LP	Novel

Het, heterozygous; Hem, hemizygote; P, pathogenic; LP, likely pathogenic; UVS, uncertain significance; E, Exon; I, Intron; ACMG, American College of Medical Genetics; MGS, mitochondrial genome sequencing; WES, whole exome sequencing; CNV, copy number variant

These variants were not frequently found in ethnically matched populations in multiple population databases; they were predicted to be damaging or deleterious, using multiple lines of prediction algorithms. Population distribution frequencies and predictive functional effects are summarized in Additional file 1: Table S3. Evolutionary conservation alignment of missense variants showed that they were highly conserved among four different species, except variants c.1612C>T p.(Arg538Trp) and c.4084A>G p.(Ile1362Val) in the *LRP5* gene (Additional file 1: Figure S3). Based on Sanger sequencing and bioinformatics analysis, we inferred that these 14 variants in the *NDP, FZD4, LRP5,* and *TSPAN12* genes were potential disease-causing variants in 13 families with FEVR.

## Discussion

In this study, we enrolled 20 probands with reduced vision who were diagnosed with FEVR based on clinical symptoms, as well as fundus and FFA examinations. We performed whole exome sequencing, Sanger sequencing validation, cosegregation analysis, functional prediction, population distribution analysis, and evolutionary conservation alignment. Our results suggested that 14 variants in the *NDP, FZD4, LRP5* and *TSPAN12* gene were potential disease-causing variants in 13 probands. Seven variants had not been reported to cause FEVR. The remaining seven families did not harbor any variant.

Clinical symptoms and fundus appearances can vary distinctly among patients and genetic backgrounds in patients with FEVR; in some instances, disease presentation can vary between eyes in a single patient [27]. For example, the proband F12 exhibited different appearances between eyes, such that the right eye demonstrated normal vision and mild fundus abnormality, while the left eye demonstrated mild reduced vision and moderate fundus abnormality. Moreover, the progress of disease was asynchronous between eyes: the right eye showed minimal progression, while the left eye showed progression with falciform retinal folds and peripheral retinal exudates at the 2-year follow-up. Distinct fundus findings were also present in proband F6, such that the right eye showed severe retinal detachment, while the left eye showed mild abnormality. Although the two probands has similar disease courses and were of similar age, their symptom severities and fundus appearances were different.

*NDP*, *FZD4*, *LRP5*, and *TSPAN12* gene variants can impair the Norrin or Wnt/β-catenin signaling pathways, which are responsible for angiopoiesis during retinal development [28]. In the canonical Wnt/β-catenin pathway, FZD4 and LRP5 form a ternary complex as a coreceptor; Wnt binds to the coreceptor and activates downstream β-catenin signaling [29]. In the Norrin/β-catenin pathway, NDP binds to the coreceptor and activates downstream β-catenin signaling with the TSPAN12 auxiliary component [30]. When these signaling pathways are activated, β-catenin translocates to the nucleus and interacts with the T-cell factor/lymphoid enhancing factor family of transcription factors, thus initiating RNA transcription and elongation [31, 32].

The *NDP* gene encodes the Norrin protein, which contains a signal peptide that directs its localization and a typical motif of six cysteines that forms a cysteine knot-like domain [33]. The variant c.118A>G p.(Met40Val) in the *NDP* gene is located in the highly conserved amino acid motif; it may affect the high affinity between Norrin and the FZD4 transmembrane protein [34].

The *FZD4* gene encodes the FZD4 protein, which contains an extracellular cysteine-rich domain, seven-pass transmembrane domains, and a frizzled domain in the extracellular region [35]. The variants c.757C>T p.(Arg253Cys), c.981G>A, p.(Trp327Ter), and c.1039T>G, p.(Phe347Val) in the *FZD4* gene are located in the transmembrane domains of the *FZD4* protein. The c.981G>A p.(Trp327Ter) variant leads to premature termination of protein translation. The other two missense variants may disturb the highly conserved region of the transmembrane domain of the FZD4 protein, leading to aberrant downstream signaling [36].

The *LRP5* gene encodes the LRP5 protein, which contains a putative signal peptide, four β-propeller motifs at the amino terminal that alternate with four epidermal growth factor-like repeats, three low-density lipoprotein receptor-like repeats, a single transmembrane domain, and a cytoplasmic domain [37]. Although the exact functions of these domains are unknown thus far, studies of LRP6 (with strong homology and similar function to LRP5) showed that the first and second β-propeller motifs, the third and fourth β-propeller motifs formed tandems to function respectively [38]. Variants located in the β-propeller motif may destroy the stable structure of β-propellers and interrupt their interactions with NDP or FZD4. The c.685C>T p.(Arg229Trp), c.1210G>A p.(Gly404Arg), c.1612C>T p.(Arg538Trp), and c.3232C>T p.(Arg1078Ter) variants in the *LRP5* gene are located in the β-propeller motif of the LRP5 protein; therefore, these variants may cause β-propeller motifs tandems to become inactive. The c.3237-2A>C variant may affect the splice mode and form a new transcript. The c.4084A>G p.(Ile1362Val) variant is located in the transmembrane domain of the LRP5 protein and may disturb the low-density lipoprotein receptor-like ligand binding domains.

The *TSPAN12* gene encodes the TSPAN12 protein, which contains four-pass transmembrane domains and four cysteines in the second extracellular region, forming two extracellular loops and an intracellular loop. Variants in transmembrane domains and extracellular regions can severely impair function, variants in the C-terminal end can moderately impair function, and variants in the N-terminal end can slightly impair function [36]. The transmembrane domains provide a scaffold for extracellular loops to change conformation and interact with FZD4 for allosteric modulation. The variants c.77T>A p.(Ile26Asn) and c.236T>G p.(Met79Arg) in the *TSPAN12* gene are located in the transmembrane domain and may disrupt the domain structure of the TSPAN12 protein, potentially preventing TSPAN12 incorporation into the receptor complex and destabilizing the NDP/FZD4/LRP5 interaction [39]. The frameshift variants c.170dupT p.(Leu57Phe fsTer60) and c.550dupA p.(Arg184Lys fsTer16) in the *TSPAN12* gene are predicted to create a premature stop codon and a truncated TSPAN12 protein.

Although we found 14 disease-causing variants, including seven novel variants, in 13 FEVR families, there were some limitations in this study. First, we only speculated that variants were potential disease-causing based on clinical manifestations, whole exome sequencing, and bioinformatics analysis. Second, the study collected a small group of samples and thus cannot expand the overall understanding of the pathogenic mechanisms by which these genes cause FEVR. We plan to validate the pathogenicity of these variants by in vivo and in vitro analyses, and we will attempt to enroll more families in a future study.

## Conclusions

In conclusion, through whole exome sequencing and bioinformatics analysis, we identified 14 variants in the *NDP, FZD4, LRP5,* and *TSPAN12* gene in 13 families with FEVR. To our knowledge, this is the first report regarding c.1039T>G p.(Phe347Val) in the *FZD4* gene; c.1612C>T p.(Arg538Trp) and c.3237-2A>C in the *LRP5* gene; and c.77T>A p.(Ile26Asn), c.170dupT p.(Leu57Phe fsTer60), c.236T>G p.(Met79Arg), and c.550dupA p.(Arg184Lys fsTer16) in the *TSPAN12* gene as potential disease-causing variants in patients with FEVR. These results expand the spectra of variants in the *NDP, FZD4, LRP5,* and *TSPAN12* genes. We presume that these findings will provide insights regarding accurate diagnosis, family genetic counseling, and future gene therapy for FEVR.

## Supplementary Information

**Additional file 1. Figure S1.** Pedigrees and cosegregation of families. **Figure S2.** Schematic representations of the genomic and protein structures of all variants. **Figure S3.** Evolutionary conservation of ten missense variants. **Table S1.** Primers used in Sanger sequencing. **Table S2.** Quality control information of probands with potential disease-causing variants. **Table S3.** Population distribution frequencies and predictive functional effects of variants.

## Data Availability

The sequencing data used and/or analyzed during the current study are available at the following URL: https://www.ncbi.nlm.nih.gov/sra/?term=PRJNA752456.

## Abbreviations

FEVR: Familial exudative vitreoretinopathy
NDP: Norrin
FZD4: Frizzled class receptor 4
LRP5: Low density lipoprotein receptor-related protein 5
TSPAN12: Tetraspanin 12
FFA: Fundus fluorescein angiography
BCVA: Best-corrected visual acuity
ACMG: American College of Medical Genetics
