# Supplementary material for: Whole exome sequencing revealed 14 variants in NDP, FZD4, LRP5, and TSPAN12 genes for 20 families with familial exudative vitreoretinopathy
Source: BMC Med Genomics. 2022 Mar 11;15:54. doi: 10.1186/s12920-022-01204-0 (PMC8915523; doi:10.1186/s12920-022-01204-0)
Supplement: Supplementary file 1 — Additional file 1. Figure S1. Pedigrees and cosegregation of families. Figure S2. Schematic representations of the genomic and protein structures of all variants. Figure S3. Evolutionary conservation of ten missense variants. Table S1. Primers used in Sanger sequencing. Table S2. Quality control information of probands with potential disease-causing variants. Table S3. Population distribution frequencies and predictive functional effects of variants. [file 12920_2022_1204_MOESM1_ESM.docx]

Supplementary Materials

Supplementary Figure 1. Pedigrees and cosegregation of families.

Supplementary Figure 2. Schematic representations of the genomic and protein structures of all variants.

Supplementary Figure 3. Evolutionary conservation of ten missense variants.

Supplementary Table 1. Primers used in Sanger sequencing.

Supplementary Table 2. Quality control information of probands with potential disease-causing variants.

Supplementary Table 3. Population distribution frequencies and predictive functional effects of variants.

Supplementary Table 1. Primers used in Sanger sequencing

| **Gene** | **Nucleotide change** | **Location** | **Primers** | **Amplicon (bp)** |
| --- | --- | --- | --- | --- |
| *NDP* | c.118A>G | ChrX:43817774 | F: GAAAGCTTCAGCCCGATCCT | 530 |
|  |  |  | R: GACACCTGTCCCACTGGATG |  |
| *FZD4* | c.757C>T | Chr11:86663041 | F: TGACCCCATTTGAGTCCTGC | 570 |
|  |  |  | R: ATTCCCACCACAGAACGACC |  |
| *FZD4* | c.981G>A | Chr11:86662817 | F: TGAACAAGGCCACCAAACCT | 504 |
|  |  |  | R: AGTTTTCCTACCCTGAGCGCC |  |
| *FZD4* | c.1039T>G | Chr11:86662759 | F: CTGTAGGCCGGGAAAGGATA | 496 |
|  |  |  | R: ACCCCAATCTTGACCATCAGT |  |
| *LRP5* | c.685C>T | Chr11:68125314 | F: TCCACAAGAAGGGAAGGTGC | 599 |
|  |  |  | R: AATGCCACACAGACCCTGAC |  |
| *LRP5* | c.1210G>A | Chr11:68153978 | F: CCATTGCACCTGTCTCCACA | 529 |
|  |  |  | R: TCCGGGTTCTGATGCAAGAC |  |
| *LRP5* | c.1612C>T | Chr11:68170978 | F: GGAACCATGCTACCTGGTGT | 524 |
|  |  |  | R: TGTCACCTTTCCTGTCCTGC |  |
| *LRP5* | c.3232C>T | Chr11:68191161 | F: CCAGTGCTCAGGAGTCTTGG | 593 |
|  |  |  | R: AGGAACAGGAGCGAGTCAACA |  |
| *LRP5* | c.3237-2A>C | Chr11:68192568 | F: CTAGTATAGAATGTGACCTGTCAGCCT | 382 |
|  |  |  | R: GACAATGAAGGCCGGGTGTCT |  |
| *LRP5* | c.4084A>G | Chr11:68204440 | F: CTGTACTAGACCACTCCCCG | 371 |
|  |  |  | R: GAGTCTCCCACACAGCCC |  |
| *TSPAN12* | c.77T>A | Chr7:120480153 | F: AGATCAAGGAAGAGCACTACCATA | 463 |
|  |  |  | R: TATTTGTTCTGTGGCCTCCTTATC |  |
| *TSPAN12* | c.170dupT | Chr7:120478945 | F: TGAGTTGGGGTAGGAAAGCT | 561 |
|  |  |  | R: CCTCATCCGGTACAGCAAAT |  |
| *TSPAN12* | c.236T>G | Chr7:120478880 | F: ACGTCTGCTACCTCACAACC | 509 |
|  |  |  | R: CCTGTGGTTCATCCGGTCAT |  |
| *TSPAN12* | c.550dupA | Chr7:120446664 | F: CCTGGTCTTTCCCACTTCCC | 540 |
|  |  |  | R: CGTTAGCCATGGTGTTCCT |  |

Supplementary Table 2. Quality control information of probands with potential disease-causing variants

| **ID** | **Raw data size (M)** | **No. of total reads** | **Mean depth of target region (X)** | **Percentage of covered target region (%)** | | **Mean depth of MGS** | **Qualified rate of Q30 (%)** |
| --- | --- | --- | --- | --- | --- | --- | --- |
|  |  |  |  | **20X** | **30X** |  |  |
| 1 | 12,593 | 125,928,734 | 139.05 | 98.95 | 97.47 | 1,609.52 | 93.73 |
| 2 | 12,780 | 127,798,916 | 147.71 | 99.42 | 98.46 | 3,113.94 | 92.35 |
| 3 | 12,360 | 123,598,596 | 138.02 | 98.76 | 97.73 | 1,806.18 | 91.47 |
| 4 | 11,257 | 102,567,543 | 115.24 | 99.56 | 98.50 | NA | 93.41 |
| 5 | 10,801 | 108,014,416 | 125.02 | 98.79 | 97.72 | 2,139.66 | 94.28 |
| 6 | 12,447 | 82,981,458 | 155.29 | 97.44 | 96.77 | NA | 93.35 |
| 7 | 11,974 | 119,743,060 | 141.93 | 99.44 | 98.55 | 3,416.61 | 91.82 |
| 8 | 11,609 | 116,086,686 | 123.69 | 99.44 | 98.21 | 3,021.57 | 91.55 |
| 9 | 13,076 | 130,759,436 | 149.34 | 99.37 | 98.56 | 3,866.08 | 92.25 |
| 10 | 10,593 | 105,925,808 | 114.62 | 99.25 | 97.57 | 1,411.51 | 90.46 |
| 11 | 16,939 | 112,931,350 | 164.55 | 99.75 | 99.54 | NA | 95.32 |
| 12 | 10,614 | 70,765,264 | 118.52 | 99.42 | 98.60 | NA | 91.50 |
| 13 | 11,563 | 115,627,218 | 129.12 | 98.83 | 97.91 | 2,133.82 | 90.82 |

Abbreviations: MGS, mitochondrial genome sequencing; NA, not available.

Supplementary Table 3. Population distribution frequencies and predictive functional effects of variants

| **ID** | **Gene** | **Variation** | **Amino acid change** | **1000 Genomes** | **ExAC** | **GnomAD (exome)** | **GnomAD (genome)** | **PROVEAN** | **PP2-**  **HDIV** | **PP2-**  **HVAR** | **Mutation**  **Taster** | **ClinPred** | **LRT** | **SIFT** | **Mutation**  **Assessor** | **FATHMM** | **GERP** |
| --- | --- | --- | --- | --- | --- | --- | --- | --- | --- | --- | --- | --- | --- | --- | --- | --- | --- |
| 1 | *NDP* | c.118A>G | p.(M40V) | NA | NA | NA | NA | -1.034 | 0.817 | 0.096 | 1.000 | 0.742 | 0.843 | 0.479 | 0.895 | NA | 5.81 |
| 2 | *FZD4* | c.757C>T | p.(R253C) | NA | 8.263E-06 | 1.194E-05 | 1.194E-05 | -7.81 | 1.000 | 1.000 | 1.000 | 0.993 | 0.843 | 0.000 | 3.090 | 0.85 | 5.69 |
| 3 | *FZD4* | c.981G>A | p.(W327X) | NA | NA | NA | NA | NA | NA | NA | 1.000 | NA | 0.843 | NA | NA | NA | 5.59 |
| 4 | *FZD4* | c.1039T>G | p.(F347V) | NA | NA | NA | NA | -6.76 | 1.000 | 0.997 | 1.000 | NA | NA | 0.000 | NA | -2.48 | NA |
| 5 | *LRP5* | c.685C>T | p.(R229W) | NA | 6.846E-05 | 4.540E-05 | NA | -6.26 | 1.000 | 0.991 | 1.000 | 0.960 | 0.460 | 0.000 | 3.455 | -4.48 | 3.76 |
| 6 | *LRP5* | c.1210G>A | p.(G404R) | NA | 8.265E-06 | 1.219E-05 | 3.233E-05 | -7.15 | 1.000 | 0.999 | 1.000 | 0.998 | 0.000 | 0.000 | 3.490 | -5.61 | 2.87 |
| 6 | *LRP5* | c.1612C>T | p.(R538W) | NA | 1.651E-05 | 8.122E-06 | NA | -5.18 | 0.034 | 0.032 | 1.000 | 0.691 | 0.463 | 0.020 | 3.750 | -4.15 | 2.05 |
| 7 | *LRP5* | c.3232C>T | p.(R1078 X) | NA | NA | NA | NA | NA | NA | NA | NA | NA | NA | NA | NA | NA | 4.80 |
| 8 | *LRP5* | c.3237-2A>C | - | NA | NA | NA | NA | NA | NA | NA | NA | NA | NA | NA | NA | NA | 5.05 |
| 9 | *LRP5* | c.4084A>G | p.(I1362V) | NA | 8.260E−06 | 6.400E-04 | 3.200E-05 | 0.44 | 0.000 | 0.004 | 0.967 | 0.075 | 0.068 | 0.198 | -0.105 | -3.70 | 4.37 |
| 10 | *TSPAN12* | c.77T>A | p.(I26N) | NA | NA | NA | NA | -2.38 | 0.178 | 0.262 | 1.000 | 0.745 | 0.296 | 0.019 | 1.320 | -1.44 | 5.66 |
| 11 | *TSPAN12* | c.170dupT | p.(L57FfsTer60) | NA | NA | NA | NA | NA | NA | NA | NA | NA | NA | NA | NA | NA | NA |
| 12 | *TSPAN12* | c.236T>G | p.(M79R) | NA | NA | NA | NA | -3.19 | 0.106 | 0.148 | 1.000 | 0.855 | 0.000 | 0.020 | 0.690 | -1.26 | 5.99 |
| 13 | *TSPAN12* | c.550dupA | p.(R184KfsTer16) | NA | NA | NA | NA | NA | NA | NA | NA | NA | NA | NA | NA | NA | NA |

Note: SIFT, Sorting Intolerant From Tolerant, it ranges from 0 to 1, deleterious (<0.05), tolerated (>=0.05), lower values are more deleterious; PROVEAN, Protein Varia tion Effect Analyzer, it ranges from -14 to 14, -14~-2.5, deleterious, -2.5~14, neutral, lower values are more deleterious; PolyPhen2 HDIV, probably damaging (>=0.957), possibly damaging (0.453-0.956), benign (<=0.452), higher values are more deleterious; PolyPhen2 HVAR, probably damaging (>=0.909), possibly damaging (0.447-0.909), benign (<=0.446), higher values are more deleterious; ClinPred, deleterious (>=0.5), tolerated (<0.5), higher values are more deleterious; LRT, Likelihood Ratio Test, lower values are more deleterious; Mutation Taster, higher values are more deleterious; Mutation Assessor, higher values are more deleterious; FATHMM, Functional Analysis Through Hidden Markov Models, lower values are more deleterious; GERP, Genomic Evolutionary Rate Profiling, it ranges from -12.3 to 6.17, higher values are more conserved and deleterious. Abbreviations: NA, not available; ExAC, Exome Aggregation Consortium database; GnomAD, Genome Aggregation database.


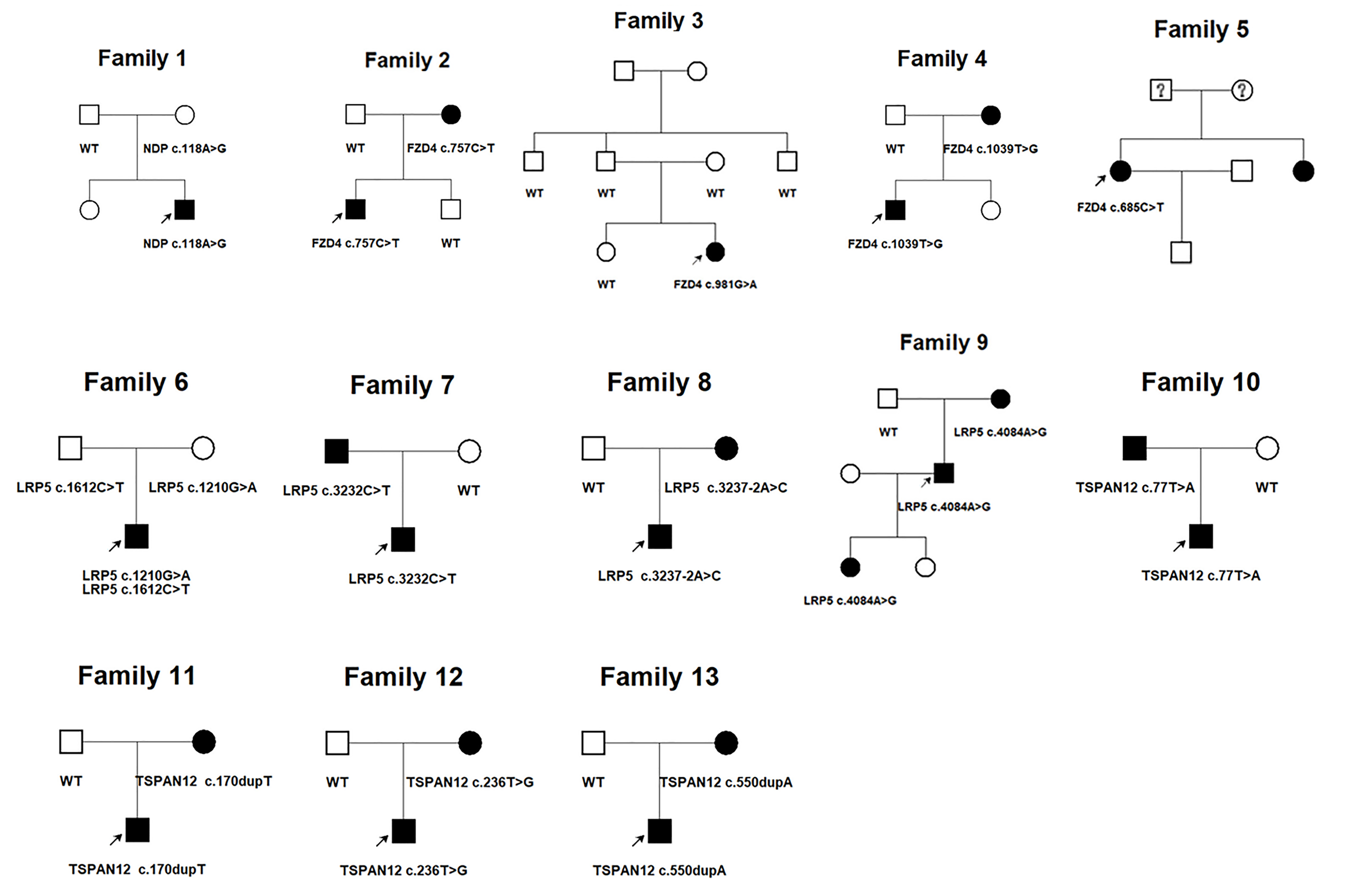


Supplementary Figure 1. Pedigrees and cosegregation of families. While variant c.981G>A in the *FZD4* gene did not cosegregate with the disease in family 3 and peripheral blood samples were unavailable for family 5, other variants were cosegregated with the phenotypes of FEVR. Circles denote unaffected females, boxes denote unaffected males, dark symbols indicate affected participants, question marks denote unknown family history, and arrows denote probands. Abbreviations: WT, wild-type.


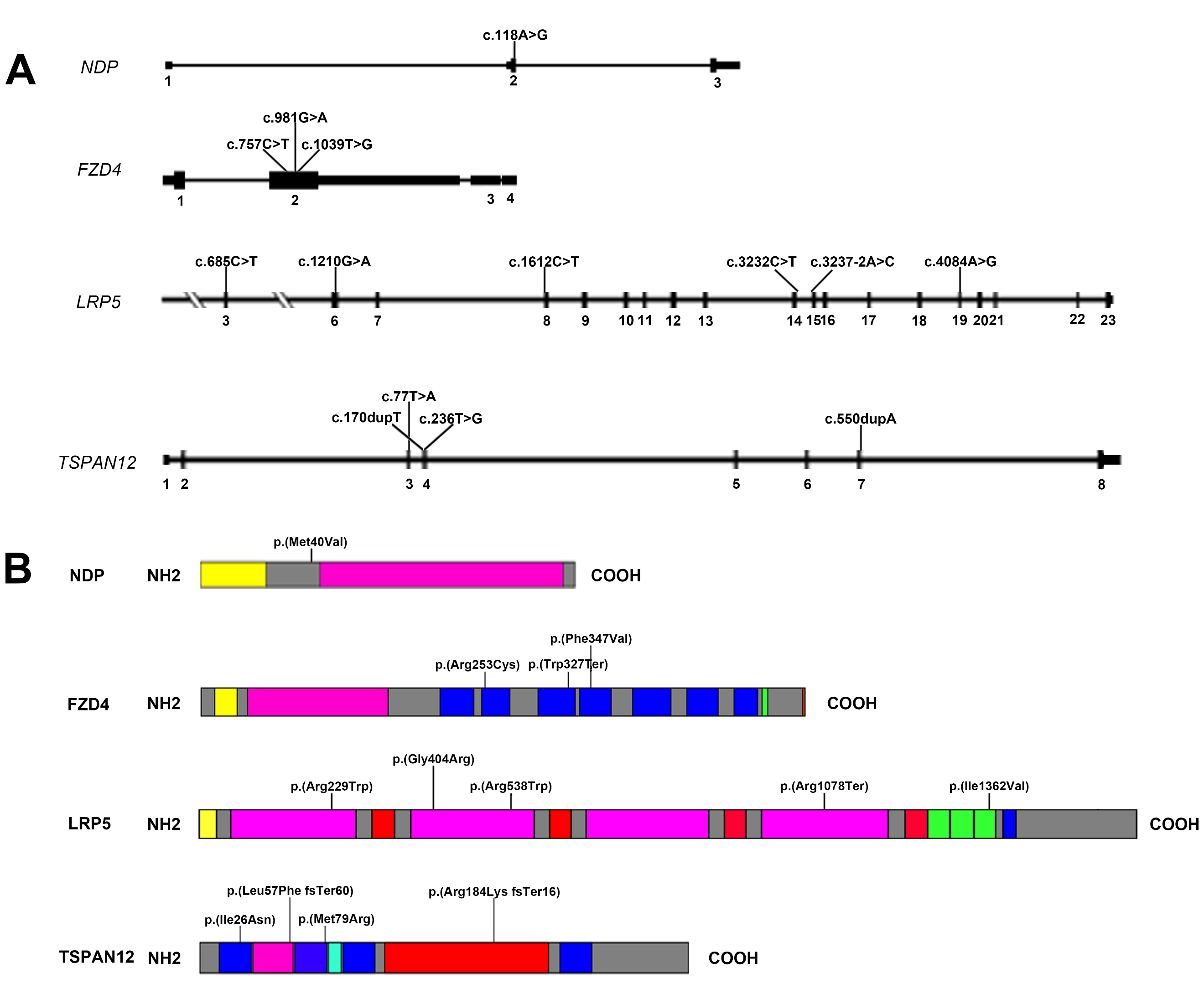


Supplementary Figure 2. A, Schematic representations of the genomic structures of all variants. Numbers below diagrams indicate corresponding exon numbers, parts of exons are omitted. B, Schematic representations of the protein structures of all variants. NDP protein: yellow band denotes signal peptide, and pink band denotes C-terminal cystine knot-like domain. FZD4 protein: yellow band denotes signal peptide, pink band denotes cystine-rich domain, blue bands denote transmembrane domains, and green band denotes Lys-Thr-X-X-X-Trp motif. LRP5 protein: yellow band denotes signal peptide, pink bands denote β-propeller motifs, red bands denote epidermal growth factor repeats, green bands denote low density lipoprotein receptor-like repeats, and blue band denotes transmembrane domain. TSPAN12 protein: pink band denotes the first (small) extracellular loop, green band denotes intracellular loop, red band denotes the second (large) extracellular loop, and blue bands denote transmembrane domains.


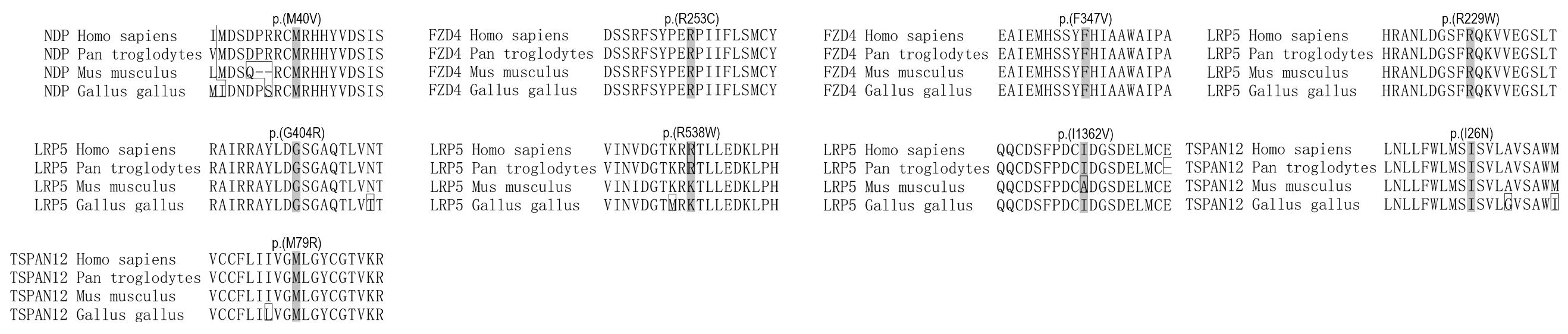


Supplementary Figure 3. Evolutionary conservation of ten missense variants. With the exceptions of variants c.1612C>T p.(Arg538Trp) and c.4084A>G p.(Ile1362Val) in the *LRP5* gene, other residues affected by missense variants in the NDP, FZD4, LRP5, and TSPAN12 proteins are highly conserved among four different species.
